# Aggregated Alpha-Synuclein Inclusions within the Nucleus Predict Impending Neuronal Cell Death in a Mouse Model of Parkinsonism

**DOI:** 10.3390/ijms232315294

**Published:** 2022-12-04

**Authors:** Leah J. Weston, Anna M. Bowman, Valerie R. Osterberg, Charles K. Meshul, Randall L. Woltjer, Vivek K. Unni

**Affiliations:** 1 Department of Neurology & Jungers Center for Neurosciences Research, Oregon Health & Science University, Portland, OR 97239, USA; 2 Research Services, Neurocytology Laboratory, Veterans Affairs Medical Center, Portland, OR 97239, USA; 3 Departments of Behavioral Neuroscience and Pathology, Oregon Health & Science University, Portland, OR 97239, USA; 4 Department of Pathology, Oregon Health & Science University, Portland, OR 97239, USA; 5 OHSU Parkinson Center, Oregon Health & Science University, Portland, OR 97239, USA

**Keywords:** Lewy pathology, nuclear inclusions, multiphoton imaging, nuclear rods

## Abstract

Alpha-synuclein (aSyn) is a 14 kD protein encoded by the SNCA gene that is expressed in vertebrates and normally localizes to presynaptic terminals and the nucleus. aSyn forms pathological intracellular aggregates that typify a group of important neurodegenerative diseases called synucleinopathies. Previous work in human tissue and model systems indicates that some of these aggregates can be intranuclear, but the significance of aSyn aggregation within the nucleus is not clear. We used a mouse model that develops aggregated aSyn nuclear inclusions. Using aSyn preformed fibril injections in GFP-tagged aSyn transgenic mice, we were able to induce the formation of nuclear aSyn inclusions and study their properties in fixed tissue and in vivo using multiphoton microscopy. In addition, we analyzed human synucleinopathy patient tissue to better understand this pathology. Our data demonstrate that nuclear aSyn inclusions may form through the transmission of aSyn between neurons, and these intranuclear aggregates bear the hallmarks of cytoplasmic Lewy pathology. Neuronal nuclear aSyn inclusions can form rod-like structures that do not contain actin, excluding them from being previously described nuclear actin rods. Longitudinal, in vivo multiphoton imaging indicates that certain morphologies of neuronal nuclear aSyn inclusions predict cell death within 14 days. Human multiple system atrophy cases contain neurons and glia with similar nuclear inclusions, but we were unable to detect such inclusions in Lewy body dementia cases. This study suggests that the dysregulation of a nuclear aSyn function associated with nuclear inclusion formation could play a role in the forms of neurodegeneration associated with synucleinopathy.

## 1. Introduction

Alpha-synuclein (aSyn) is a small 14 kD protein encoded by the SNCA gene that is expressed in vertebrate neurons and localizes to presynaptic terminals and the nucleus [1]. aSyn is stable in a monomeric or potentially tetrameric conformation [2,3]; however, it can form pathological intracellular aggregates that are tightly associated with a group of neurodegenerative diseases often known as synucleinopathies. Synucleinopathies impact over 10 million people worldwide and include multiple diseases that have a range of symptoms, such as motor deficits in Parkinson’s disease (PD), dementia in Lewy Body dementia (LBD) and PD dementia (PDD), and autonomic failure and motor deficits in multiple system atrophy (MSA) [4,5,6]. In PD and LBD, aSyn aggregates are found in inclusions in the soma and processes of neurons. MSA is characterized by glial cytoplasmic inclusions (GCIs) found predominantly in oligodendrocytes, with less frequent glial nuclear and neuronal cytoplasmic and nuclear inclusions. The staining of neuronal and glial aSyn inclusions with congophilic dyes, such as thioflavin S and X-34, confirms that they are made of a fibrillar species in an amyloid conformation, and antibody staining reveals that these aggregates contain high levels of aSyn serine-129 phosphorylation (pSyn). Recently developed propagation models in rodents suggest that this aggregation is seeded by small aSyn fibrillar species that spread in a prion-like manner via trans-synaptic pathways [7,8,9,10]. Understanding this aSyn inclusion formation and spread is critical for elucidating the mechanisms underlying synucleinopathies and could lead to the development of new disease-modifying treatments.

aSyn functions at the presynaptic terminal, where it can interact with the SNARE complex and participate in neurotransmitter vesicle cycling [11]; however, it also has underappreciated roles in the nucleus. Despite early suggestions of its localization to the nucleus, the presence of nuclear aSyn has been controversial due to the existence of nonspecific staining when certain antibodies are used [1,12]. Recently, however, multiple different groups and approaches have confirmed that aSyn can localize to the nucleus and its function there is still being unraveled [13,14,15,16]. Evidence suggests that nuclear aSyn can have normal physiologic functions, including regulating gene transcription [15], participating in nucleocytoplasmic transport [17], and facilitating DNA double-strand break repair [13]. Other evidence suggests that nuclear aSyn can also, at times, be detrimental. Increased nuclear aSyn due to post-translational modifications such as serine-129 phosphorylation or familial PD-associated point mutations have been suggested to cause toxicity in vitro [16,18,19]. aSyn also plays a role in epigenetic regulation through its interactions with the DNA methylation machinery and histones, and these interactions have been shown to increase neurotoxicity [20,21]. Although the role of aSyn in the nucleus is an increasing area of interest, there is still little known about the potential role of aSyn nuclear inclusions in disease.

Pathological aSyn nuclear inclusions have been most often described in MSA, but there are also reports of certain kinds of nuclear aggregates being present in PDD and LBD, suggesting a potentially more widespread involvement of nuclear aggregates in many synucleinopathies [5,14,16]. Autopsy studies suggest that glial nuclear inclusions (GNIs) and neuronal nuclear inclusions (NNIs) are found in about 80% of MSA patients [22]. Both types of nuclear inclusions are ubiquitinated, pSyn positive, and composed of fibrillar filaments that are 10–20 nm in diameter [23,24]. GNIs are almost always found in GCI-positive cells [23]; however, the percent of GCI-positive cells with nuclear inclusions varies widely from study to study. In contrast, NNIs are also described in cells without corresponding cytoplasmic inclusions, leading to the idea that these inclusions may develop in the early stages of disease, before the formation of neuronal cytoplasmic inclusions (NCIs) [22,24]. In addition, a correlation has been found between the number of NNIs and the number of surviving neurons in the pons and inferior olivary nucleus [22]. Even with this previous work, the role of NNIs and GNIs in disease progression and cellular health remains largely unexplored, and our understanding would greatly benefit from the development of model systems where this can be studied in more detail.

Although there are reports of nuclear inclusions in multiple model systems, the focus of many of these studies has been on the associated cytoplasmic pathology. For instance, an early transgenic PD mouse model expressing human aSyn under control of the PDGF promoter reported dense nuclear inclusions; however, the characteristics of these inclusions were not extensively described [25]. We have also briefly reported nuclear inclusions in a transgenic human aSyn-expressing mouse model after injection with aSyn preformed fibrils (PFFs) in the cortex [26]. Additionally, wildtype mice that received a gastric wall PFF injection displayed nuclear inclusions [27]. The formation of nuclear inclusions increases when specific aSyn PFF strains are used or if toxin insults, such as MPTP exposure, are combined with PFF injection [28,29]. In vitro models can also display nuclear inclusions. Cell cultures expressing aSyn tagged with a nuclear localization signal develop pSyn-positive nuclear inclusions, and this aggregation is not caused by the localization signal [16]. Primary neuron cultures treated with fibrils formed from copper-exposed aSyn showed increased nuclear inclusions, which is consistent with mouse data that indicate that specific aSyn strains have a greater propensity for nuclear aggregation [28,30]. Following neurotoxin exposure, apoptotic cells in culture increase their nuclear aSyn and exhibit nuclear aSyn aggregation [31]. The morphology of nuclear inclusions can vary widely, ranging from small “dot”-like structures at the limit of light resolution to larger aggregates with multiple shapes, but the significance of these morphological differences is not clear [27,29]. Furthermore, the effect of nuclear inclusions on cellular processes in these model systems is poorly understood.

In this study we aimed to better understand the role of nuclear inclusions in disease pathology using a mouse model that develops nuclear inclusions and an analysis of synucleinopathy patient tissue. Using aSyn PFF injections in GFP-tagged aSyn (Syn-GFP) transgenic mice, we were able to induce the formation of nuclear aSyn inclusions and study their fixed tissue and in vivo properties. To examine the relevance of nuclear inclusions in human disease, MSA and LBD brain tissue was screened for four types of inclusions, NCIs, NNIs, GCIs, and GNIs, and compared to our mouse model.

## 2. Results

### 2.1. Neuronal Nuclear Inclusions Develop in Mouse Models

**Figure 1:**
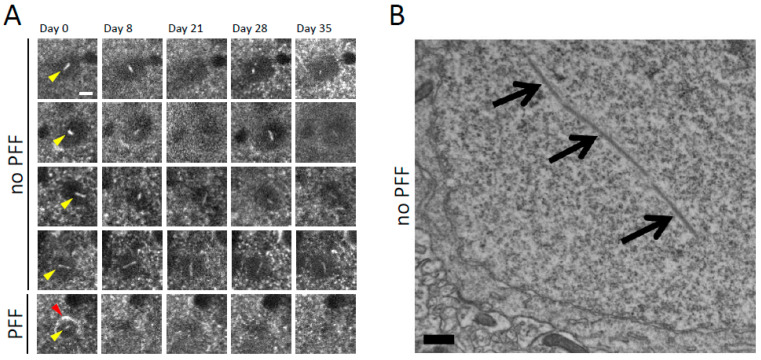
Intranuclear alpha-synuclein-GFP rod-like aggregates form spontaneously in transgenic mouse cortical neurons and in neurons bearing somatic Lewy pathology after PFF injection. (**A**) *Top rows:* In vivo multiphoton imaging performed serially over 5 weeks shows four different examples (each row a different cell) of mouse cortical neurons in Syn-GFP transgenic animals bearing intranuclear Syn-GFP rod-like aggregates (yellow arrowheads). Intranuclear aggregates are mobile within the nucleus at this time scale, changing orientation within the nucleus over the course of days. *Bottom row:* In vivo imaging from a cortical neuron bearing a somatic Syn-GFP Lewy inclusion (red arrowhead) induced by alpha-synuclein PFF injection, also with an intranuclear aggregate (yellow arrowhead), on day 0 of imaging, which degenerates and disappears before day 8. Scale bar: 5 μm. (**B**) Electron micrograph of cortical neuron in Syn-GFP transgenic animal (with no PFF injection) demonstrating a rod-like intranuclear aggregate (arrows). Scale bar: 500 nm.

We used in vivo multiphoton imaging of layer 2/3 of mouse sensory-motor cortex via a “cranial window,” with approaches we have previously developed, to study the presence and properties of nuclear aSyn inclusions [8,10,26,32]. The mouse line used expresses wildtype human sequence aSyn tagged with GFP on its C-terminus under the control of the PDGF promoter in about 2–3% of cortical neurons [32]. Although soluble Syn-GFP is present in the cytoplasm and nucleoplasm in expressing cells, our previous work, which used a combination of in vivo fluorescence recovery after photobleaching (FRAP) approaches and staining for molecular markers in fixed tissue, shows that most cells in this model contain somatic and nuclear Syn-GFP that is freely diffusible and not aggregated [33]. This mouse line does develop Syn-GFP aggregates in presynaptic terminals, which are associated with synaptic dysfunction [33], but frank neuronal cell death is not observed [10]. In this study, we noticed that a small percentage of Syn-GFP-expressing cells developed rod-like Syn-GFP structures, and that based on their positioning within the soma and ability to rotate within the cell over the course of days were located within the nucleus (Figure 1A). Electron microscopic analysis of cortical tissue from these Syn-GFP animals also demonstrated the presence of linear structures within the nucleus that were not present in nontransgenic animals (Figure 1B). As we previously reported, aSyn PFF injection into these Syn-GFP animals induces the formation of cytoplasmic aSyn aggregates that are composed of Syn-GFP, which are present in amyloid conformation and have post-translational modifications typical of human Lewy pathology as assayed using a combination of approaches. These approaches include immunohistochemistry, X-34 and thioflavin S staining, in vivo FRAP, and correlated light-electron microscopy [8,10,26]. In addition to cytoplasmic inclusions, satellite inclusions within the nucleus can also develop in neurons with pre-existing somatic Lewy inclusions [26]. We again detected the development of these NNIs using our in vivo imaging approaches (Figure 1A) and have attempted to better characterize their properties in this study.

### 2.2. Nuclear Inclusions Composed of Transgenic Syn-GFP Can Occur in Cells That Do Not Express Syn-GFP

**Figure 2:**
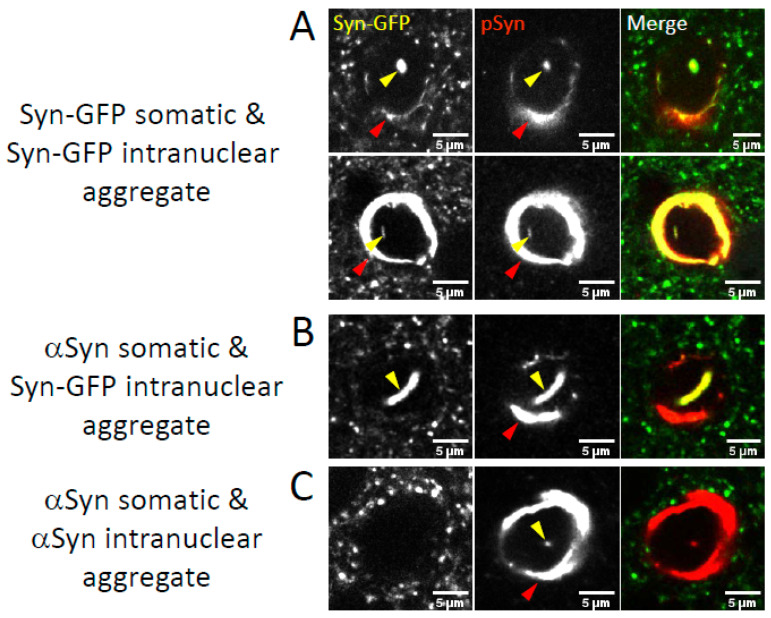
Intranuclear aggregates form in neurons bearing somatic Lewy pathology after PFF injection. Confocal imaging in fixed tissue from PFF-injected Syn-GFP transgenic animals induced to form Lewy pathology shows examples of inclusions where both the somatic (red arrowheads) and intranuclear (yellow arrowheads) aggregates are Syn-GFP positive (**A**), where the somatic inclusion is made up of endogenous mouse alpha-synuclein (not transgenic Syn-GFP), but the intranuclear aggregate is Syn-GFP positive (**B**), and where both the somatic and intranuclear aggregates are made up of endogenous mouse alpha-synuclein (**C**). All aggregates are also stained for the marker of alpha-synuclein aggregation serine-129 phosphorylation (pSyn). Scale bar: 5 μm.

Alpha-synuclein in a pathologic conformation is highly phosphorylated at serine-129, and this pSyn is an established marker of Lewy pathology in human tissue [34]. We used pSyn staining in PFF-injected fixed tissue paired with endogenous GFP fluorescence from the Syn-GFP protein to determine the presence of NNIs in cortical neurons with Syn-GFP-positive cytoplasmic Lewy pathology versus those with cytoplasmic Lewy pathology made up of only the endogenous (nonfluorescent) mouse aSyn. We detected neurons where both the cytoplasmic and nuclear inclusions in the same cell were positive for Syn-GFP and pSyn (Figure 2A) and those where both cytoplasmic and nuclear inclusions were Syn-GFP negative and pSyn positive (composed of only endogenous mouse aSyn, Figure 2C). Interestingly, we also rarely detected the presence of NNIs which were both Syn-GFP and pSyn positive, but the cytoplasmic inclusion within that cell was Syn-GFP negative (and pSyn positive, Figure 2B). There are several possible explanations for this observation, but since this mouse line only expresses Syn-GFP in 2–3% of cortical neurons [32], one possibility is that NNIs may form from aSyn that is transmitted into a neuron from other cells. If this is the case, Syn-GFP is being transported into neurons that do not express it, where it accumulates in the nucleus of these recipient cells. It will be interesting to test in future work if this transport into the nucleus may be associated with the parallel or subsequent formation of a cytoplasmic inclusion in the recipient cell.

### 2.3. Neuronal Nuclear Inclusions Bear the Hallmarks of Genuine Lewy Pathology

**Figure 3:**
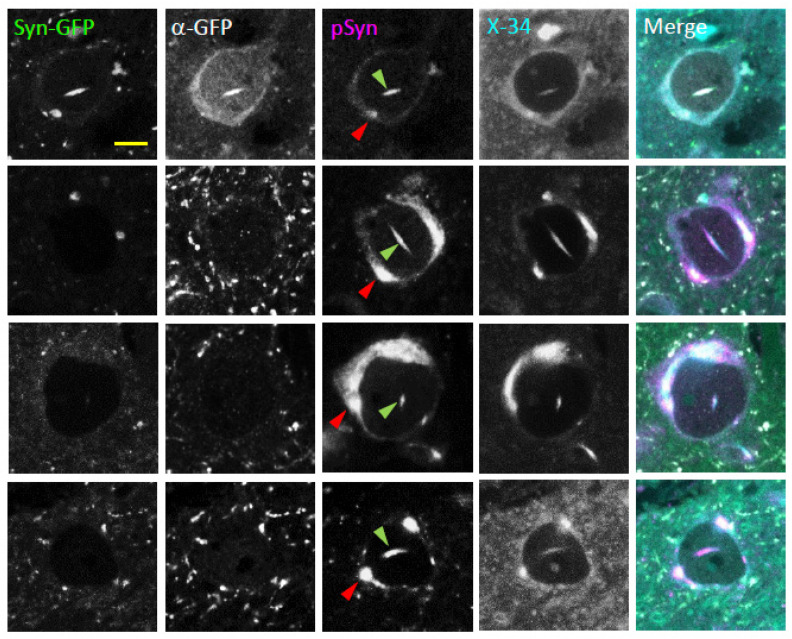
Intranuclear alpha-synuclein aggregates in neurons bearing somatic Lewy pathology are phosphorylated at serine-129 and in an amyloid fibril state. Confocal imaging in fixed tissue from PFF-injected Syn-GFP transgenic animals induced to form Lewy pathology shows four examples of somatic (red arrowhead) and intranuclear (green arrowhead) inclusions (each row a different cell) imaged for endogenous GFP fluorescence (Syn-GFP), with an anti-GFP antibody (α-GFP), with a serine-129 phospho-synuclein antibody (pSyn), and for fluorescence from the dye X-34 that labels fibrillar proteins in an amyloid conformation. **Top** row: Syn-GFP-expressing neuron. **Bottom** three rows: Syn-GFP-negative neurons only expressing endogenous mouse alpha-synuclein. Scale bar: 5 μm.

To better understand the molecular properties of NNIs, we stained both Syn-GFP-positive and -negative inclusions in fixed tissue with an antibody to GFP to confirm that the endogenous Syn-GFP signal colocalized with the anti-GFP antibody staining as expected (Figure 3). All inclusions, both Syn-GFP positive and negative, stained positive for the Lewy pathology marker pSyn (Figure 3). In addition, we stained fixed tissue with the small molecule dye X-34, a derivative of Congo red, which labels fibrils with a cross beta-pleated sheet structure and stains aSyn aggregates with a higher signal-to-noise ratio than thioflavin S in our mouse model [10,35]. This dye selectively binds a molecular structure where the direction of the beta-pleated sheet is perpendicular to the long axis of the fibril, also known as an amyloid conformation. This amyloid conformation is typical of aSyn in Lewy pathology. Like the cytoplasmic Lewy pathology within the same cell, NNIs are also labeled by X-34 (Figure 3). This pattern of NNIs staining positive for both pSyn and X-34 indicates that NNIs in this model share several of the important molecular properties of aggregated aSyn found within human Lewy pathology.

### 2.4. Neuronal Nuclear Inclusions Are Not Composed of Actin

**Figure 4:**
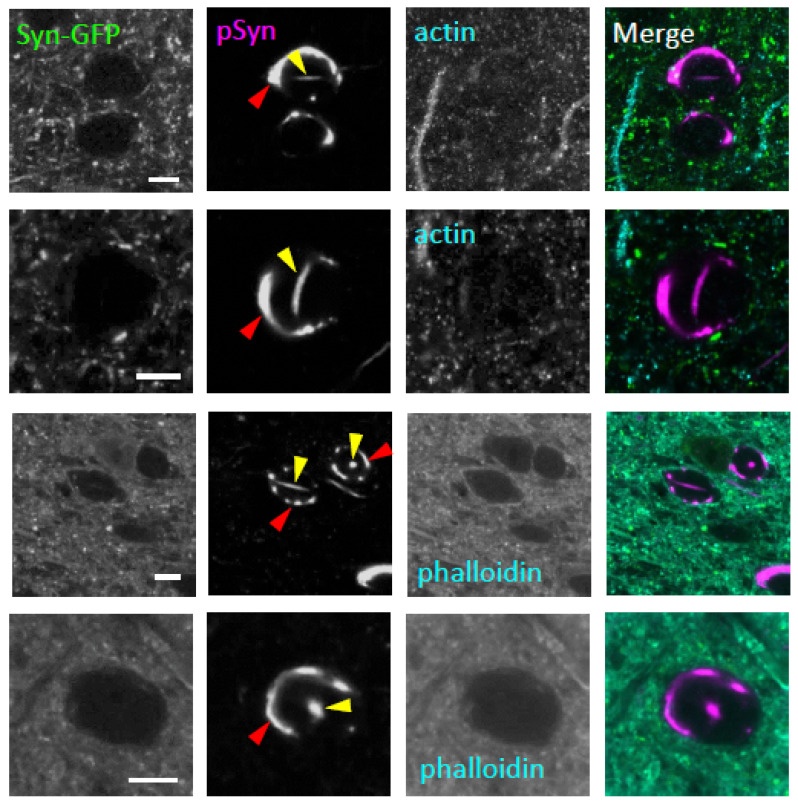
Intranuclear alpha-synuclein aggregates in neurons bearing somatic Lewy pathology do not have an actin component. Confocal imaging in fixed tissue from PFF-injected Syn-GFP transgenic animals induced to form Lewy pathology shows four examples of somatic (red arrowhead) and intranuclear (yellow arrowhead) inclusions (each row a different cell) imaged for endogenous GFP fluorescence (Syn-GFP), with a serine-129 phospho-synuclein antibody (pSyn), an actin-specific antibody (*top two rows*), and the actin-binding dye phalloidin (*bottom two rows*). Scale bars: 5 μm.

Pathological brain tissue from several neurodegenerative diseases has been shown to contain linear inclusions composed of cofilin and actin that have been called “actin rods.” Although these actin rods are found in the cytoplasm in most diseases where they are described, actin rods have been reported within the nucleus in Huntington’s disease [36]. To test whether the aSyn NNIs that we detected in our PFF-injection Syn-GFP mouse model were composed of actin and, therefore, similar to the actin rods seen in Huntington’s disease, we measured the endogenous GFP fluorescence and stained with antibodies for pSyn and actin in fixed cortical tissue. Although we detected prominent nuclear, rod-shaped pSyn-positive inclusions in neurons with cytoplasmic inclusions, these nuclear inclusions did not stain positive for actin (Figure 4). Next, we similarly stained tissue with the heptapeptide dye phalloidin, which labels filamentous actin, including actin rods, in other neurodegenerative diseases. In our system, however, aSyn NNIs that were linear in shape or with other morphologies were always negative for phalloidin staining (Figure 4). This staining pattern suggests that aSyn NNIs in neurons with cytoplasmic Lewy pathology are not an example of the actin rods seen in Huntington’s disease.

### 2.5. The Presence of Certain Neuronal Nuclear Inclusions Can Predict Imminent Cell Death

**Figure 5:**
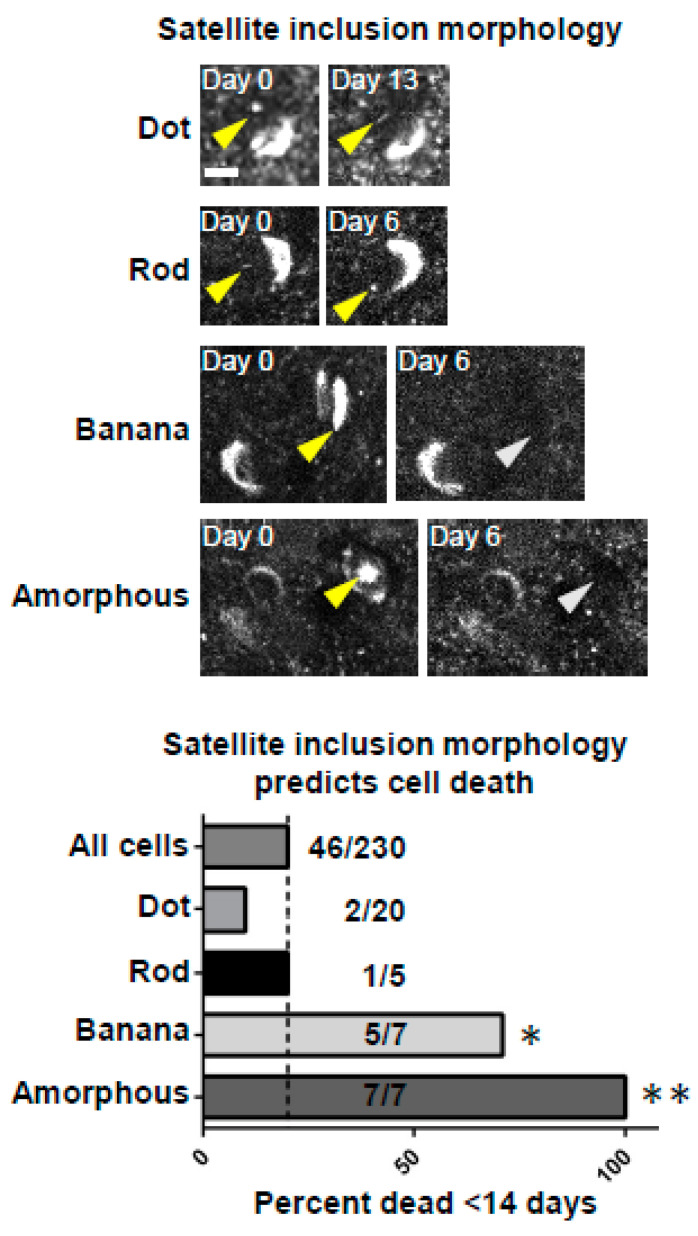
Development of specific intranuclear aggregate morphologies predicts imminent cell death. **Top***:* Examples of specific intranuclear aggregate morphologies (dot, rod, banana, and amorphous). Scale bar: 5 μm. **Bottom***:* Group data showing predictive value for the development of intranuclear inclusions with different morphologies in predicting cell death within the next 14 days compared to the frequency expected from “all cells”, which includes those with and without intranuclear inclusions (dotted line). The first value to the right of each column represents the number of cells that died within 14 days in that category, and the second number represents the total number of cells detected in that category. The probability of observing this number of cell death events by chance using the binomial distribution predicted *p*-values * < 0.005 for “banana” and ** <0.0001 for “amorphous” inclusions.

To understand how NNIs could relate to neurodegeneration within neurons that develop them, we used in vivo multiphoton imaging to longitudinally follow cortical neurons bearing somatic inclusions with and without detectible nuclear inclusions. Interestingly, this analysis of Lewy inclusion-bearing neurons in vivo over time suggests that specifically shaped aggregates of aSyn could predict upcoming cell death. Our previous work in this model suggested that when looking at all neurons that develop somatic Syn-GFP Lewy inclusions, they have a half-life of ~6 months after inclusion formation [10,26]. In the current analysis, we suspected that the development of a nuclear inclusion with a specific morphology could predict cell death in a much shorter time frame than 6 months. To test this, we analyzed all Lewy inclusion-bearing cell death events in our longitudinal in vivo imaging data, including in neurons with and without nuclear inclusions. We categorized the NNIs into four different specific morphologies and conducted a blinded analysis of all neurons that degenerated in <14 days and compared this fraction to the fraction bearing NNIs that died in <14 days. This analysis demonstrated that NNIs with specific morphologies could predict imminent cell death of that neuron within <14 days (Figure 5). These data suggest that the presence of NNIs within neurons bearing somatic Lewy inclusions is a poor prognostic factor for that cell and that degeneration could be related to the dysregulation of nuclear processes that trigger programmed cell death.

### 2.6. Human Multiple System Atrophy Neurons and Glia Contain Nuclear Inclusions

**Figure 6:**
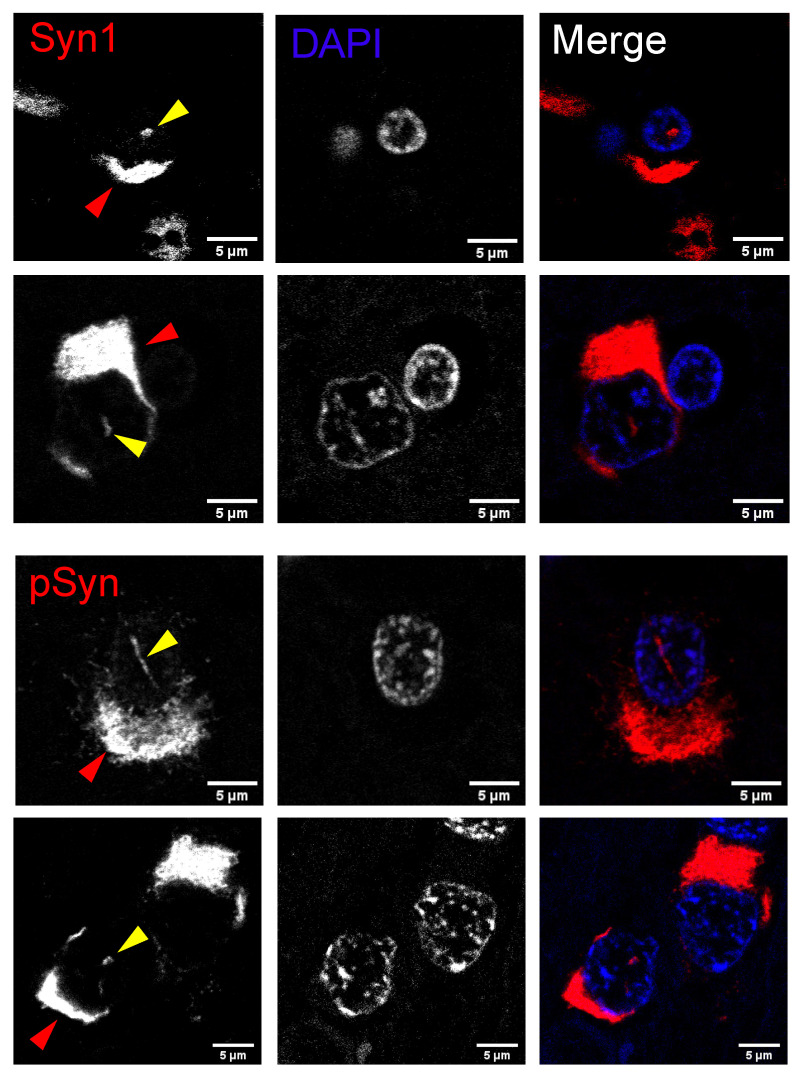
Intranuclear alpha-synuclein aggregates are present in pontine neurons and glia bearing somatic synuclein pathology in human cases of multiple system atrophy. Confocal imaging in fixed tissue from the pons in human cases of multiple system atrophy shows examples of intranuclear alpha-synuclein aggregates (yellow arrowhead) labeled with a total alpha-synuclein antibody (Syn1, top two rows) or a serine-129 phospho-synuclein antibody (pSyn, bottom two rows). Intranuclear aggregates are in glia (top row) and neurons (bottom three rows), as defined by morphological criteria, and in cells that also contain cytoplasmic pathology (red arrowhead). Scale bar: 5 μm.

aSyn nuclear inclusions are a well-defined characteristic of MSA [22,23], but there are also reports of aggregated nuclear aSyn in other synucleinopathies [14,16]. To determine the pathological relevance of nuclear inclusions in synucleinopathies, we screened LBD and MSA tissue for cytoplasmic and nuclear aSyn inclusions in glial cells and neurons. The majority of LBD and MSA cases displayed both GCIs and NCIs; however, in our dataset only MSA cases showed nuclear inclusions. In the MSA cases, 90% exhibited NNIs and 50% contained GNIs (Table 1). We further characterized MSA nuclear inclusions by labeling brain areas with a high NNI density, the putamen and pons, for aSyn and pSyn. Nuclear inclusions were observed in both glia and neurons (Figure 6). The majority of nuclear inclusions were observed in cells also bearing cytoplasmic inclusions. These nuclear inclusions were positive for pSyn and aSyn (Figure 6). Furthermore, NNIs displayed multiple morphologies, such as long rod-like conformations and dot-like inclusions (Figure 6), suggesting that the morphological patterns seen in our mouse model are similarly present in human MSA cases.

## 3. Discussion

We have confirmed the presence of nuclear inclusions composed of aggregated aSyn both in human MSA and mouse model brain tissue. In our Syn-GFP mouse line, linear, mobile, rod-shaped Syn-GFP nuclear aggregates form spontaneously in a small proportion of neurons even without the induction of Lewy pathology, but this has no obvious consequence in terms of cell viability that we can detect. Further work will be required to determine if these spontaneously forming intranuclear rods contribute to specific alterations in cellular function or not. In contrast, aSyn PFF-induced NNIs are correlated with imminent cell death. These NNIs are in a subset of cells also containing cytoplasmic Lewy inclusions, induced to form by aSyn PFF injection. These NNIs in PFF-injected brain are not composed of actin, making them unlikely to be actin rods, but are composed of aSyn aggregated in an amyloid conformation and phosphorylated at serine-129, two hallmarks of Lewy pathology. In our study we did not test the proteinase K sensitivity of the intranuclear aSyn inclusions detected, neither in the mouse model nor MSA patient tissue. It will be informative in future work to determine whether these intranuclear inclusions are resistant to proteinase K, as has been described for somatic aSyn inclusions in MSA [37], and whether they contain other aSyn post-translational modifications that have been variably associated with Lewy pathology in human cases, such as truncation, ubiquitination, nitration, and other oxidative changes. Interestingly, our data suggest that there may be transmission of aggregated aSyn protein from one cell to the nucleus of another cell, since Syn-GFP-positive NNIs can be detected in neurons bearing somatic inclusions composed of endogenous (untagged) mouse aSyn. Additionally, the presence of NNIs with specific morphologies is highly predictive of impending neuronal death, potentially indicating that the dysregulation of a nuclear aSyn function could play an important role in neurodegeneration.

One of the more intriguing observations from our data in PFF-injected mouse brain transgenically expressing Syn-GFP in a small subset of cortical neurons is the rare presence of NNIs composed of Syn-GFP in a neuron containing a cytoplasmic inclusion that is Syn-GFP negative. One possible explanation for this observation is that a trans-cellular transport of aSyn, potentially in an aggregated form, can occur between neurons and this involves the movement of aSyn out of one cell and into the cytoplasm and then nucleus of another cell. Although there is ample evidence that aSyn can be secreted and taken up by neurons in culture, and a leading model of PFF-induced Lewy pathology formation and spread in mouse brain suggests the trans-synaptic transport of a small aggregated aSyn “seed” species between neurons, to our knowledge, there is no previous data suggesting that aSyn internalized by a cell is delivered to its nucleus. There is elegant work, however, demonstrating that certain homeobox transcription factors can be secreted by cells and contain activity that allows them to translocate across the neuronal plasma membrane in a receptor-independent manner [38]. These homeoproteins accumulate in the neuron’s nucleus and exert their neurotrophic activity via DNA binding [39,40,41,42]. The Engrailed homeoprotein has also been specifically suggested to use this mechanism to maintain dopamine neuron survival in the midbrain by reducing the ability of mobile genetic elements to induce DNA strand breaks [43,44]. The compromise of this activity can lead to a PD-like phenotype in mice [43]. It will be important to test in future work whether aSyn may have similar properties, allowing it to be taken up by cells and transported into the nucleus. Here, or on its way to the nucleus, it could potentially seed the formation of fibrillar aSyn from protein already in the cell and, thus, trigger cytoplasmic inclusion formation.

A second, unexpected observation from our data is that the presence of NNIs with specific morphologies can predict upcoming neuronal death within a period of 14 days. Our previous work showed that neurons bearing cytoplasmic Lewy inclusions in this model have a half-life of 6 months or more, suggesting that neurons can live with these cytoplasmic inclusions for relatively long periods of time [10,26]. However, the presence of specific nuclear inclusions with curvilinear (“banana”) or amorphous shapes strongly predicted upcoming cellular demise. Whether there is something particular about these specific shapes, or if they just represent forms of NNIs which have relatively large volumes, is unclear. Regardless, their presence is tightly associated with cell death within a short time frame and this suggests that there may be the dysregulation of nuclear processes associated with these NNIs that could lead to the induction of apoptosis or other programmed cell death pathways. Our own previous work implicates aSyn in the repair of nuclear DNA damage, and others have suggested that aSyn can modulate transcription and histone modification, making it a possibility that one or more of these candidate processes could be dysregulated [13,20,21]. It will be important in future work to develop assays to monitor these processes in neurons containing specific NNIs to test whether these pathways could be dysregulated when nuclear inclusions form.

MSA has long been described as a neurodegenerative disorder with nuclear inclusions. Papp and Lantos suggested that the formation of NNIs occurred early, before NCI formation, and even before aSyn was known to be the primary component making up these inclusions [45,46]. Later work using aSyn immunohistochemistry also suggested that NNIs do precede NCIs, and that NNIs can sometimes form in association with the nucleolus within the nucleus [22,23]. Our data suggest that nuclear inclusions, either NNIs or GNIs, are found relatively exclusively in human cases in MSA, since we were not able to detect nuclear inclusions in LBD cases. This is consistent with previous reports using traditional immunohistochemical techniques and showing nuclear inclusions in MSA, but not LBD [47]. In contrast, others have reported the presence of other forms of aSyn aggregates in LBD using different approaches, including subcellular fractionation to biochemically measure smaller aggregate species that are not easily detected by immunohistochemistry [14]. This suggests that although different forms of intranuclear aSyn aggregation may occur in multiple synucleinopathies, MSA is the only one where nuclear inclusions can be detected by wide-field or confocal microscopy and immunohistochemistry. It will be important to test whether the microscopically observable intranuclear inclusions detected in neurons and glia in MSA represent a directly toxic form, or are rather simply a marker of the presence of other smaller, more difficult to observe oligomeric aSyn species that are directly mediating toxicity. These smaller, intranuclear, oligomeric aSyn species have been suggested to occur in LBD as well [14], and may, therefore, represent a common neurodegenerative pathway relevant to both MSA and LBD.

Our data suggest that aSyn nuclear inclusions are present in PFF mouse models of synucleinopathy and that they may play important roles in disease pathobiology. These nuclear inclusions are composed of serine-129 phosphorylated and aggregated aSyn in a fibrillar amyloid form that can potentially be transmitted between cells. Our in vivo multiphoton imaging data demonstrate that the presence of these nuclear inclusions in neurons predicts their impending cell death and suggest that the dysregulation of a nuclear aSyn function could play a role in this neurodegeneration. Further work should focus on better characterizing the molecular nature of these intranuclear inclusions, the processes that lead to their formation, and testing whether they are causally related to cell death. A deeper understanding of the mechanisms of nuclear aSyn aggregation and its consequences for brain cells could define new targets for therapeutic intervention in several important neurodegenerative diseases.

## 4. Methods and Materials

### 4.1. Animals

Animals were housed by OHSU’s Department of Comparative Medicine in a light–dark cycle, temperature and humidity-controlled vivarium, and maintained under an ad libitum food and water diet. All experiments were approved by the OHSU IACUC. All experiments were performed in accordance with the relevant guidelines and regulations, and every effort was made to minimize the number of animals used and their distress. Syn-GFP animals have been previously described (PDNG78 [32]).

### 4.2. Lewy Pathology Formation

Two- to three-month-old mice were injected with mouse wildtype sequence PFFs (gift from Kelvin Luk, University of Pennsylvania, Philadelphia, PA, USA). Injections were performed according to published protocols for PFF generation, preparation, and cortical injections [7,10]. Briefly, 2.5 μL (2 mg/mL) of freshly sonicated PFFs was injected into the right hemisphere primary sensory-motor cortex in isoflurane (1% to 2%)-anesthetized animals.

### 4.3. Mouse Tissue Immunohistochemistry

Immunohistochemistry was carried out similarly to previously published protocols [26,33]. Fixed hemispheres were mounted on a Leica VT1000 S Vibratome and sagittally sectioned into 50 μm slices. Primary antibodies (serine-129 phospho-alpha-synuclein: 1:667, 81A, BioLegend, San Diego, CA, USA; GFP: 1:3000, ab6556, Abcam, Cambridge, UK; actin: 1:100, A0483, Sigma-Aldrich, St. Louis, MO, USA) were incubated with shaking in the dark overnight at 4 °C. Tissue was washed in PBS five times for 30 min each time. Secondary antibodies, goat anti-mouse or goat anti-rabbit Alexa Fluor 647 (Invitrogen, Waltham, MA, USA), were diluted to 1:2000 and subjected to incubated shaking in the dark overnight at 4 °C. Tissue was washed in PBS five times for 30 min each time at room temperature. X-34 staining was performed using standard techniques and was provided as a gift from William Klunk (University of Pittsburgh, Pittsburgh, PA, USA) [35]. Phalloidin staining was performed using the manufacturer’s protocols (Alexa Fluor 555 Phalloidin, Fisher Scientific, Waltham, MA, USA). For confocal imaging, images were acquired on a Zeiss Elyra PS.1 confocal microscope with a Plan-Apochromat 63×/1.40 oil objective.

### 4.4. Mouse Tissue Electron Microscopy

Electron microscopy (EM) was carried out similarly to previously published protocols [33]. Animals were perfused using a transcardiac approach with 6 mL of 1000 units/mL of heparin in a 0.1 M phosphate buffer (pH 7.3), which was then immediately followed with 50 mL of 0.5% paraformaldehyde/2.5% glutaraldehyde/0.1% picric acid in a 0.1 m phosphate buffer at a pH of 7.3 and at room temperature. The primary sensory and motor cortex was processed for EM using a microwave procedure as described in [33]. Thin sections (60 nm) were cut on an ultramicrotome (EM UC7; Leica Microsystems, Deerfield, IL, USA) along the leading edge of the cortical tissue block, where layers I-VI were exposed, using a diamond knife (Diatome, Hatfield, PA, USA). Photographs were taken on a JEOL 1400 transmission electron microscope.

### 4.5. In vivo Multiphoton Imaging

Cranial window surgery, imaging, and analysis were conducted using a similar protocol as we have previously published for isoflurane-anesthetized animals at 4–5 months of age (2 months after PFF injection) [10,26,33]. We used a Zeiss LSM 7 MP multiphoton microscope outfitted with dual-channel BiG (binary GaAsP) detectors and a Coherent Technologies Chameleon titanium-sapphire femtosecond pulsed laser source (tuned to 860 nm for imaging Syn-GFP). Zeiss Zen 2011 image acquisition software was used.

### 4.6. Humans

Human subjects were seen in the Oregon Alzheimer’s Disease Center (OADC) and affected subjects had an established clinical diagnosis. Brain tissue from autopsies of ten patients diagnosed with Lewy body dementia and ten patients with multiple system atrophy was procured in a de-identified manner from the OADC neuropathology core. Tissue use was approved by the IRB at OHSU.

### 4.7. Human Tissue Immunohistochemistry

Ten MSA and ten LBD cases were provided through the OADC neuropathology core. Standard histological methods were used to evaluate aSyn expression in the frontal gyrus, hippocampus, amygdala, and midbrain for all cases, and in the putamen and pons in MSA cases. Briefly, formalin-fixed, paraffin-embedded (FFPE) tissue sections were incubated with Syn1 (1:1000; Fisher Scientific, Waltham, MA, USA) or EP1536Y (1:5000; Abcam, Cambridge, UK), developed with diaminobenzidine (DAB) chromagen, and counterstained with hematoxylin. Additionally, MSA cases were immunofluorescently labeled, and 7 μm FFPE sections from the pons and putamen were blocked in 5% BSA in PBS + 1% TritonX-100 (PBST) for 1 hr at room temperature before incubation overnight at 4 °C in EP1536Y (1:500; Abcam, Cambridge, UK) or Syn1 (1:500; Fisher Scientific, Waltham, MA, USA) diluted in 1% BSA PBST. Appropriate secondary antibodies (Alexa 555; 1:1000; Abcam, Cambridge, UK) and DAPI diluted in 1% BSA PBST were incubated for 1 hr at room temperature. Lipofuscin autofluorescence was quenched in 1× TrueBlack Plus (Biotium, Fremont, CA, USA). Immunohistochemical and immunofluorescent slides were imaged on a Zeiss ApoTome2 microscope and Zeiss LSM 980 confocal microscope, respectively.

## Figures and Tables

**Table 1:** Analysis of human MSA and LBD brain tissue for different types of inclusions. A series of 10 MSA and 10 LBD cases were examined the presence (✓) or absence of alpha-synuclein aggregates in different subcellular locations and cell types. Abbreviations: glial cytoplasmic inclusion (GCI), glial nuclear inclusion (GNI), Lewy body dementia (LBD), multiple system atrophy (MSA), neuronal cytoplasmic inclusion (NCI), neuronal nuclear inclusion (NNI).

MSA	Age	Sex	GCI	NCI	NNI	GNI
Case 1	69	m	✓	✓	✓	
Case 2	70	f	✓	✓	✓	✓
Case 3	68	f	✓	✓	✓	
Case 4	73	m	✓	✓	✓	
Case 5	64	m	✓	✓	✓	✓
Case 6	65	f	✓	✓	✓	✓
Case 7	75	f	✓	✓	✓	✓
Case 8	68	m	✓			✓
Case 9	72	m	✓	✓	✓	
Case 10	72	f	✓	✓	✓	
**LBD**	**Age**	**Sex**	**GCI**	**NCI**	**NNI**	**GNI**
Case 1	40	f	✓	✓		
Case 2	99	f		✓		
Case 3	76	m	✓	✓		
Case 4	81	m		✓		
Case 5	73	m	✓	✓		
Case 6	102	f	✓	✓		
Case 7	71	m	✓	✓		
Case 8	74	m	✓	✓		
Case 9	73	m	✓	✓		
Case 10	74	m	✓	✓		

## Data Availability

The data presented in this study are available in the article or on request from the corresponding author.
